# Biocatalysis by Transglutaminases: A Review of Biotechnological Applications

**DOI:** 10.3390/mi9110562

**Published:** 2018-10-31

**Authors:** Maria Pia Savoca, Elisa Tonoli, Adeola G. Atobatele, Elisabetta A. M. Verderio

**Affiliations:** School of Science and Technology, Interdisciplinary Biomedical Research Centre, Nottingham Trent University, Nottingham NG11 8NS, UK; maria.savoca2016@my.ntu.ac.uk (M.P.S.); elisa.tonoli2015@my.ntu.ac.uk (E.T.); adeola.atobatele022014@my.ntu.ac.uk (A.G.A.)

**Keywords:** transglutaminases, crosslinking, polymerisation, food industry, biomedicine

## Abstract

The biocatalytic activity of transglutaminases (TGs) leads to the synthesis of new covalent isopeptide bonds (crosslinks) between peptide-bound glutamine and lysine residues, but also the transamidation of primary amines to glutamine residues, which ultimately can result into protein polymerisation. Operating with a cysteine/histidine/aspartic acid (Cys/His/Asp) catalytic triad, TGs induce the post-translational modification of proteins at both physiological and pathological conditions (e.g., accumulation of matrices in tissue fibrosis). Because of the disparate biotechnological applications, this large family of protein-remodelling enzymes have stimulated an escalation of interest. In the past 50 years, both mammalian and microbial TGs polymerising activity has been exploited in the food industry for the improvement of aliments’ quality, texture, and nutritive value, other than to enhance the food appearance and increased marketability. At the same time, the ability of TGs to crosslink extracellular matrix proteins, like collagen, as well as synthetic biopolymers, has led to multiple applications in biomedicine, such as the production of biocompatible scaffolds and hydrogels for tissue engineering and drug delivery, or DNA-protein bio-conjugation and antibody functionalisation. Here, we summarise the most recent advances in the field, focusing on the utilisation of TGs-mediated protein multimerisation in biotechnological and bioengineering applications.

## 1. Transglutaminases: Enzymatic Activity and Regulation

Mammalian transglutaminases (TGs) have been extensively characterised in the past 60 years since their discovery by Heinrich Waelsch in 1957 [1]. They constitute a family of eight catalytically active acyl-transferases (TG1-7 and factor XIIIa), plus the inactive erythrocyte protein band 4.2 (EPB4.2) [2]. TGs are mostly known for the ability to catalyse the formation of intra- and inter-molecular covalent bonds between proteins, also referred to as crosslinking activity [3]. The presence of ε-(γ-glutamyl)lysine crosslinks was first reported in human [4] and bovine [5] fibrin polymerised by FXIIIa. Over the years, it has been confirmed that also the other members of the TG family are capable of protein crosslinking, with the exception of EPB4.2, a catalytically inactive form [6,7,8].

**Figure 1:**
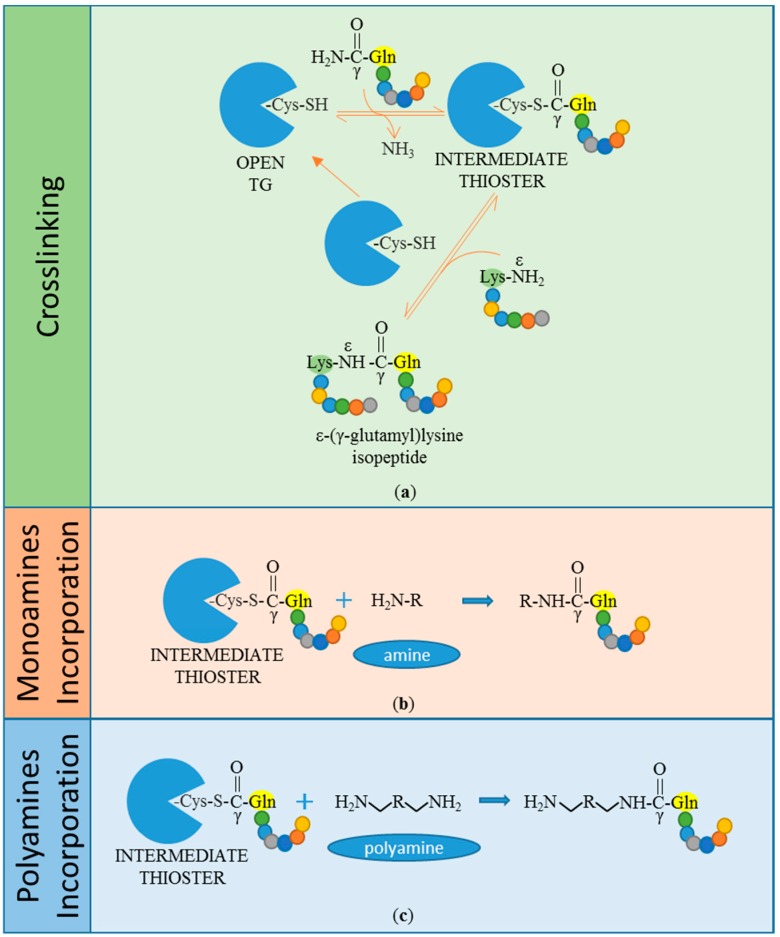
Transamidation reactions catalysed by transglutaminases (TGs). In response to triggering Ca^2+^ concentration and in appropriate redox conditions, TG conformation is open and the catalytic cysteine (Cys) thiol (SH) group is prone to bind the γ-carboxamide group of a peptide-bound glutamine residue (Gln). Therefore, a thioester bond is created between the TG’s Cys and the Gln of a peptide target, with consequent ammonia release (**a**). TG catalyses the transfer of the acyl intermediate product to a nucleophilic substrate, like an ε-amino group of a peptide-bound lysine residue (Lys), leading to the formation of ε-(γ-glutamyl)lysine isopeptide bond, also called a crosslink (**a**). TG catalyses the incorporation of monoamines (**b**) or polyamines (**c**), acting as acyl-acceptors in a reaction similar to the crosslinking.

The crosslinking reaction occurs in two consecutive steps: At first, an intermediate thioester is formed through the attack of an acyl donor (γ-carboxamide group of a peptide-bound glutamine residue) by the nucleophilic active thiolate (cysteine residue), with consequent release of ammonia. Secondly, the thiolate is restored by nucleophilic attack of an acyl acceptor substrate (ε-amino group of a peptide-bound lysine residue) (Figure 1a). This leads to the formation of a covalent inter-molecular ε-(γ-glutamyl)lysine isopeptide bond, which is resistant to physical and chemical degradation [3,9,10,11]. A similar reaction leads to the incorporation of primary amines, including polyamines, into the γ-carboxamide group of peptide-bound glutamine (Gln) residues (Figure 1b,c). Both reactions are calcium-dependent and together are referred to as protein transamidation. Notably, the transamidation reaction, which leads to amine incorporation, was actually the first to be identified by Waelsch and colleagues in 1957, by the detection of radiolabelled transamidated polyamines in guinea pig liver protein extracts [1,12].

**Figure 2:**
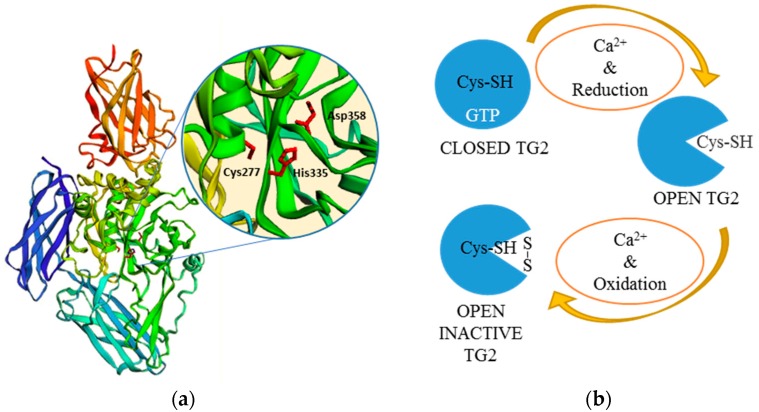
Human transglutaminase 2 (hTG2) structure and regulation. (**a**) hTG2 structure is shown in closed conformation. The catalytic site is composed by the triad, Cys277, His335, and Asp358. In this conformation, the protein is inactive, since the two β-barrels hide the catalytic pocket. Three-dimensional structure (PDB: 4PYG) was produced with the molecule modelling software, “EzMol” (version 1.22) [34]. (**b**) Effect of redox regulation on TG2 conformations. TG2 is locked in closed conformation when bound to guanosine triphosphate (GTP) and calcium concentration is low. Conversely, it assumes an open conformation after calcium binding, which can either be inactive when in oxidising conditions or active in reducing conditions [35].

Among mammalian TGs, tissue transglutaminase (tTG) or transglutaminase 2 (TG2) (NM_004613.2) has been by far the most studied, mainly because of its diverse proprieties and involvement in multiple physiological and pathological processes. TG2 is composed by 687 amino acids (aa), it is ubiquitously expressed in several different cell types and, like the other active members, is defined by its calcium-dependent transamidating activity [13,14]. TG2 structure consists of four globular domains (Figure 2a). The core domain (aa 140–460), key for the transamidation activity, is characterised by the catalytic triad, cysteine-histidine-aspartic acid (Cys277-His335-Asp358) [15,16], plus two tryptophan residues (W241 and W332), which stabilise the reaction intermediate product [17,18]. The N-terminal β-sandwich domain (aa 1–139) includes the binding site for fibronectin (FN) [19,20,21], while the two C-terminal β-barrel domains (aa 461–586 and 587–687) are involved with the TG2 ability to bind and hydrolyse guanosine/adenosine triphosphate (GTP/ATP) [16,22,23,24]. TG2 undergoes an allosteric activation fostered by calcium (Ca^2+^) with a dissociation constant (K_d_) of 90 μmol·L^−1^ [25]. Seeing that the TG2-Ca^2+^ bound X-ray structure is not available, the crystal structures of other TGs (i.e., TG3 and FXIIIa) and computational homology-based three-dimensional models of TG2 have been used to study the Ca^2+^ binding sites [26,27]. Out of the six Ca^2+^ binding sites that have been identified, five influence enzymatic activity and act in a cooperative manner. In physiological conditions (Ca^2+^: 0.1 μmol·L^−1^/GTP: 100–150 μmol·L^−1^), intracellular TG2 is completely inhibited. It is believed that TG2 inhibition is mainly accomplished by guanine nucleotides, i.e., GTP, guanosine diphosphate (GDP), guanosine monophosphate (GMP), and adenine nucleotides (ATP) [22,25,28]. When Ca^2+^ levels are sufficiently increased (0.5–1.5 mmol·L^−1^), GTP inhibitory capability is significantly reduced, likely due to the conformational changes caused by Ca^2+^ binding [16,28,29,30]. Additionally, other molecules, such as heparan sulfate moieties of proteoglycans (HSPG), may influence TG conformation [31]. Besides TG2, also other members of the TGs family (TG3, 5 and 6) have been reported to be inhibited by purine nucleotides, with different responsiveness levels [22,28,32,33].

Calcium and purine nucleotides are not the only regulators of TGs. In particular, the redox state affects the accessibility of the Cys active site and it is also essential for TGs’ crosslinking activity [35,36,37,38]. Recent knowledge suggests that TG2 can assume three conformations: An inactive form bound to GTP, an inactive one bound to Ca^2+^, but oxidised, and a reduced one activated by Ca^2+^ [38] (Figure 2b). Under reducing conditions, Ca^2+^ binding decreasing TGs’ affinity for GTP/GDP leads to an enzymatically active “open” conformation [38,39,40], while GTP binding causes the “closed” conformation, blocking substrate access to the catalytic pocket [39,40]. Experimental data confirm that transamidation is not only dependent on the Ca^2+^/GTP ratio. In fact, extracellular TG2 is mostly inactive even when the low GTP/Ca^2+^ ratio would theoretically promote activation, at least until induced by a chemical or physical injury [41]. This might be explained by TG2 being predominantly locked in closed conformation, possibly due to the redox state of the extracellular environment; however, other molecules may also further modulate TG conformation (e.g., HSPG, integrins) [31,42]. The formation of protein disulphide bridges between Cys370-Cys371 and Cys370-Cys230 in oxidising conditions is in fact sufficient to inactivate TG2 enzymatic activity [35]; conversely, reducing events cause its activation [35,38].

Besides protein transamidation, TGs are characterised by numerous other enzymatic activities. In the presence of water, TG2 is also able to hydrolyse target glutamine residues, thereby converting them into glutamic acid residues (deamidation) [43]. By deamidating gluten peptides and generating immunogenic epitopes, TG2 is responsible for the gluten-induced enteropathy celiac disease (CD) [44,45]. Additional TGs functions, such as GTPase and ATPase activity [24,28,46], protein kinase activity [47,48,49,50], and protein disulphide isomerase activity [51,52], have also been reported.

Research on TGs has led to the identification of TG homologous proteins in several species, from microorganisms to plants and animals [1,53,54]. In silico studies have allowed the identification of multiple conserved motifs in the TGs catalytic core in archaea, bacteria, and eukaryotes [55]. Conversely, the highest variability among these domains is present in the insert regions localised between the conserved motifs [55]. These studies confirmed the theory that genes codifying for TGs are derived from a unique ancestor gene expressing a cysteine protease, which then gave rise to two lineages through successive gene duplication events [55,56,57]. Specifically, one lineage includes orthologue genes from the majority of mammal TGs (TG2, TG3, TG5, TG6, TG7, and erythrocyte band 4.2), while the second one comprises the genes from invertebrates TGs, mammal TG1, and factor XIIIA [56,57].

**Figure 3:**
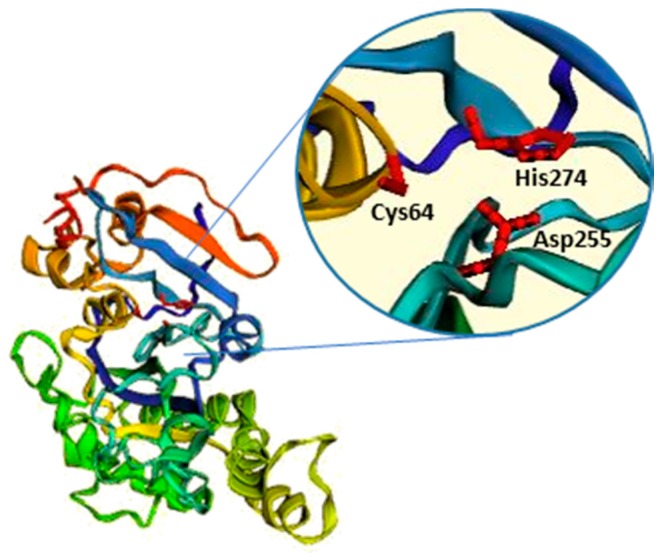
Microbial TG structure. mTG is composed by a single, compact domain. The amino acids of the active site (Cys64, Asp255, and His274) constitute the mTG catalytic triad. The modelling software, “EzMol”, was used to generate the structure (PDB: 1IU4) [34].

Among the bacterial TGs, the most relevant is microbial transglutaminase (mTG), which was first isolated from the culture medium of *Streptomyces mobarensis* and characterised by Ando and colleagues in 1989 [58,59]. mTG is a monomeric protein of about 38 kDa, consisting of 331 aa [53] and, differently from eukaryotic TGs, it is characterised by a Ca^2+^-independent crosslinking activity [58]. The overall sequence data and crystal structure indicate that mTG catalytic activity is dependent on a cysteine residue (Cys64), which, together with the adjacent Asp255 and His274 residues, overlaps well with the catalytic triad, “Cys-His-Asp”, that characterises cysteine proteases and factor XIII-like TGs [60] (Figure 3). Regulation of mTG crosslinking activity is quite different from that of mammalian TGs. For instance, mTG is not dependent on Ca^2+^, while it presents sensitivity to other cations, such as Cu^2+^, Zn^2+^, Pb^2+^, and Li^+^ [58,59].

## 2. TG2-Mediated Polymerisation of Extracellular Matrix Proteins

Among the molecules that are most likely target of TG2-induced multimerisation there are proteins found in the extracellular matrix (ECM). TG2 modifies these proteins through crosslinking, with an impact on overall matrix stabilisation/stiffness. The increased complexity of the ECM leads to increased cell-matrix interactions and changes in cell adhesion and migration [20,61,62,63,64]. FN is also a well-known target of TG2 crosslinking activity and, together with other ECM proteins (osteonectin, osteopontin, laminin, vitronectin, fibrinogen, and collagen), has been shown to be polymerised by extracellular TG2 in vitro and in various cell systems [63,64,65,66,67,68,69,70,71]. In vivo, TG2 transamidation of ECM proteins leads to their stabilisation and to the accumulation of polymeric complexes rich in isopeptide bonds, which are resistant to degradation by matrix metalloproteinases [9,72,73,74]. This results in the generation of a pathological matrix typical of fibrotic conditions, such as in kidney [72,75,76,77], lung [78,79,80,81], liver [82,83,84], and heart fibrosis [85,86,87]. Notably, skeletal phenotyping in TGs double knockout mice (Tgm2−/− and F13a1−/−) revealed that both TGs possess a synergistic function in maintaining bone mass, as their absence increases osteoclastogenesis and bone resorption. The authors also reported an increased expression of TG1 during osteoclastogenesis in both wild type and double null mice bone marrow MSCs, suggestive of a role of TG1 in osteoclast formation [88].

Hitomi’s group has developed important probes for the identification of specific substrates of TG family members, with applications in liver and kidney disease [84,89]. The mechanism of externalisation of TG2 from cells to reach the ECM is an unconventional pathway that has fascinated many research groups. Different theories have been proposed, including TG2 loading into recycling endosomes [90], TG2 secretion via purinergic P2X7 receptor-mediated vesicle shedding [91,92], cell-surface trafficking via HSPG [93,94,95], and secretion via exosomes [96,97,98].

Furthermore, TG2 itself has been shown to act as a structural adhesive protein. For example, by interacting directly with FN through a specific binding site localised in the N-terminal β-sandwich domain, TG2 forms adhesive complexes and induces cell adhesion via cell surface HSPG (syndecan-4) independently from the classic RGD-dependent cell adhesion to integrin receptors [99,100]. At the same time, it has been reported to act as an integrin co-receptor reinforcing integrin-dependent cell adhesion [42]. Several research groups have shown an interest in studying the novel TG2-HS interaction [31,94,101]. Teesalu and colleagues initially identified two sites localised in TG2 catalytic domain (aa 202–215 and aa 261–274) possibly involved in heparin binding, also suggesting that the second one could theoretically compete with FN for the binding of cell surface HS proteoglycans [101]. Soon after, Wang et al. suggested that Lys205 and Arg209 residues, which are accessible on the TG2 surface, are essential for heparin binding [94]. Two positively charged clusters (aa 262–265 and aa 598–602) have been determined by Lortat-Jacob et al. as crucial for HS binding [31]. Interestingly, these distant clusters were shown to be in spatial proximity only when TG2 is in closed conformation, which is necessary for the formation of the high affinity heparin binding domain [31].

Gaining a deeper understanding of the TG2/ECM interplay has been extremely useful for the development of several practical applications, such as the production of crosslinked matrices or for tissue engineering, which will be addressed in the following sections.

## 3. Substrate Specificity of TGs Isozymes: mTG, TG2, and FXIIIa

Since their identification in 1957, TG2 and the other members of the TGs family have shown to catalyse the modification of a variety of substrates both in vivo and in vitro. Furthermore, they have displayed a preference for the recognition of their target proteins, revealing that the transamidating reactions may be restricted to specific consensus sequences. In search for a clarification of the physiological and pathological significance of the TG transamidation, many groups have focused their research on the identification of each isozyme substrates’ specificities. 

Cousson et al. have proposed the minimal requirements for TG2-dependent modification of a putative Gln side chain in a substrate protein: (i) The residue should be accessible, either by being exposed to the solvent or located in a highly flexible region of the protein; and (ii) the amino acid sequence around the Gln should allow the correct interaction with TG, which is mainly dependent on the amino acids’ charge. Specifically, it has been shown that positively charged residues on the C-terminal side of the target Gln discourage TG catalysis [102]. Therefore, the localisation of the target amino acids is one of the main limiting factors for TG interaction, other than the protein conformation. 

Seminal work by Hitomi’s group screened potential TGs peptide substrates by creating a random peptide library by M13 phage-display. In particular, they analysed TG2 and Factor XIIIa substrates by the incorporation of biotin-labelled primary amine on phage clones expressed peptides. Among these, the following specific amino acid sequences were highlighted: QxPϕD(P), QxPϕ, and QxxϕDP for TG2; and QxxϕxWP for FXIIIa (where “x” stands for any amino acid and “ϕ” for any hydrophobic amino acid) [103]. Furthermore, a phage-display based study by Fesus’ group identified Gln-donor substrates from a random heptapeptide library by binding to recombinant TG2 and consecutive elution with a synthetic amine-donor substrate. Among these, twenty-six substrates were successfully transamidated by TG2, especially the peptides GQQQTPY, GLQQASV, and WQTPMNS. This study also confirmed pQX(P,T,S)l (where “p” stands for any polar amino acid and “l” for any aliphatic amino acid) as a consensus sequence recognised by TG2 [104]. Recently, Malešević et al. used a fluorescence-based array of tripeptides and determined that mTG could specifically recognise X-Q-Q and L-Q-X peptides (where “X” is any amino acid), with a higher preference for Y-Q-R. They also analysed mTG substrate preference in relation to amino acids adjacent to the target, Gln, highlighting a relevance for hydrophobic residues at Gln+1 and Gln-1 positions. They identified other preferred amino acids, such as tyrosine and proline in position Gln-1, but not Gln+1, while arginine presence in the tripeptides gave the opposite effect, making them poor substrates for mTG transamidation [105]. Finally, using a small focused synthetic peptide library, the tetrapeptide, “TQGA”, was identified as a novel highly specific substrate of mTG [106]. The necessity to open an active network on this topic led to the creation of TRANSDAB wiki (http://genomics.dote.hu/wiki/index.php/Main_Page/), a database that lists about 350 substrates for six human transglutaminases and mTG, along with additional interaction partners [107]. However, with the event of genomics and proteomics, we expect the number of substrates to have increased since the last update of the database was in 2010. In parallel, the database, TRANSIT (http://bioinformatica.isa.cnr.it/TRANSIT/), was generated to assess possible substrates by analysing their amino acid sequence [108]. Research in this field is ongoing, as a better knowledge of TGs specific substrates would further clarify their role in both physiology and disease. Furthermore, multiple TGs transamidation substrates have been exploited in a variety of applications, such as assay systems for in situ visualisation of TG activity [109], identification of endogenous targets of TGs in cells and tissues [84,89,110], and TGs-mediated bio-conjugation of proteins. Hence, a strong interest in finding novel and more specific TGs substrates is still alive in this area of research.

## 4. TGs Crosslinking Activity in Biotechnological Applications

Currently, the use of enzymes in biotechnological production processes is highly preferred by many biotech industries because of their wide variety and competitiveness in terms of production time and costs. The enzyme-driven crosslinking mechanism leads to changes in proteins’ hydrophobicity, thus interfering with their solubility and other properties, such as gelation, emulsification, foaming, viscosity, and water-holding capacity [111,112,113,114]. In this context, TGs have become a very popular tool for the development of different applications, an overview of which is given in Table 1.

### 4.1. Applications in Food Industry

Since the 1980s, to improve the quality and nutritive value of food, research has focused on the potential use of TGs in food processing, exploiting their ability to catalyse intermolecular isopeptide bonds and polymerise proteins [115,116,117]. Hence, different groups begun investigating the best substrates for TGs activity in this area and many were identified, such as dairy proteins (e.g., caseins and whey proteins) [118,119,120], soybean globulins [121], wheat (gluten) [122,123], myosins [124,125], egg [126], and seafood proteins [127,128].

One of the most widespread applications for TGs crosslinking activity in the food industry is the restructuring of meat and seafood by treatment of chopped muscle pieces with mTG and the polymerisation of muscle proteins (myofibrillar protein and myosin) to increase the textural characteristics and quality of the products [129,130,131,132,133,134]. Notably, mTG is not only exploited in meat processing, but also for the manufacture of dairy and bakery products, because of its considerable potential in improving the firmness, flavour, colour, texture, viscosity, elasticity, and water-binding capacity of aliments. In particular, mTG treatment during yogurt preparation was shown to improve the gel-forming properties of caseins by intermolecular crosslinking, increasing the yogurt’s breaking strength and texture, which, for example, has been applied in the production of low-fat yogurt [135,136,137]. Concerning bakery products, mTG and also guinea pig liver TG (gplTG) have been widely used to ameliorate bread and dough rheological proprieties (e.g., elasticity, stability, and volume) and shelf life [138,139,140,141,142].

A safety concern on the treatment of bakery products by TGs emerged because of the well-known involvement of TG2 and TG6 in CD [44,143]. Initial controversial studies have suggested that mTG-treated wheat and gluten-free breads increase IgA reactivity in few CD patients’ sera [144], while more recent studies showed the opposite [145]. Moreover, Heil et al. demonstrated that standard concentrations of mTG in bakery preparations (2–8 mTG units/Kg of flour) have no impact on CD incidence, even though it is not possible to exclude that higher doses might be correlated with it [145,146].

mTG cross-linking activity has also been explored for other applications, such as the preparation of chitosan-whey proteins edible films [147,148] and fish gelatin films [149,150,151,152]. The enzyme action was shown to significantly increase the films’ mechanical properties and improve other characteristics, like deformability and biodegradability [147,151]. Moreover, gplTG has been used to perform an uncommon TG-mediated glycosylation between fish gelatin hydrolysates and glucosamine, and the resulting glycopeptides had enhanced bioactivity, with a significant potential as antioxidants and antimicrobial agents [153].

Therefore, the relevance of TGs biocatalysis in the food industry continues to be explored.

### 4.2. Applications in Science and Biomedicine

TGs-mediated biocatalysis for biomedical applications has grown over the years. In this respect, TGs have raised interest because they can substitute, as non-toxic crosslinkers, the chemical agents commonly used to produce scaffold biomaterials (e.g., glutaraldehyde and formaldehyde), which leave toxic residues that are difficult to remove.

#### 4.2.1. Hydrogels and Scaffolds

One of the main applications of TGs as biocatalysts is in skin tissue engineering, for their ability to polymerise ECM proteins, especially collagens, for the production of hydrogels and scaffolds. 

Collagens are structural proteins naturally present in all vertebrates, with the important physiological function of maintaining the mechanical proprieties and integrity of connective tissues. Their characteristics of high tensile strength, high water solubility, low antigenicity, and good compatibility make them optimal to be used as biomaterials in the skin engineering sector [154]. Moreover, collagen-made matrices are known to support cell proliferation and infiltration [155,156], and thus are ideal for applications in wound-healing. However, collagens are also prone to rapid enzymatic degradation in vivo by collagenases and lack mechanical strength at high temperatures or when solubilised in aqueous media, hence they need to be stabilised by covalent crosslinks to be able to form stable structures. Various chemical crosslinking agents have been investigated, e.g., glutaraldehyde [157,158], which, however, have been shown to significantly reduce the solubility, antigenicity, and biodegradation of the collagen matrices in vitro and in vivo, presenting also some cytotoxic effects [159,160]. Several studies have been carried out to determine the chemical and physical proprieties of TG-derived collagen hydrogels as an alternative to the chemically produced ones [161,162,163]. TGs are known to induce intermolecular crosslinks in collagen fibrils [70] and to covalently bind collagen to other ECM proteins, such as FN [164]. Stachel and colleagues have shown that mTG is able to potentially create up to 5.4 crosslinks per monomer of type I collagen under denaturing conditions. When collagen is in its native conformation, half of the target Gln residues are hidden within the triple helix region of the protein, and thus are not accessible to TG catalysis, explaining why the crosslinks are efficiently created only after collagen denaturation at high temperatures [165]. In addition, TG2 and mTG biocatalysis has been exploited to incorporate polyamines and crosslink different collagens to form matrices and scaffolds [166,167,168], increasing their denaturation temperature, resistance to proteolysis, and biocompatibility, other than presenting the advantage of avoiding toxic leftovers [73,74,169,170]. Collagen-based matrices are able to enhance cell attachment, spreading, differentiation, and proliferation, as demonstrated in dermal fibroblasts and also mesenchymal stem cells (MSCs), with relevance to tissue and cartilage bioengineering [73,167]. Notably, TG2 overexpression in mammalian cell lines (human osteoblasts, endothelial cells, and mouse fibroblasts) was shown to enhance biological recognition of polymers, such as poly(DL lactide co-glycolide) (PLG), poly(e-caprolactone) (PCL), and poly(L lactide) (PLA), consequently, increasing cell attachment and spreading [171].

More recently, the suitable concentration of mTG (40 units/g) has been established for the creation of new collagen-based hydrogels, with a focus on collagenases’ degradation time [172]. Moreover, in vitro cell attachment together with in vivo biodegradability and biocompatibility assays have confirmed the high potentiality of these biomaterials in tissue engineering [172,173].

Furthermore, the use of guinea pig TG2 to crosslink amniotic membrane (AM), a scaffold employed in regenerative medicine, was shown to improve the mechanical properties of the membrane without altering the visual transparency and biocompatibility. These are fundamental features for ocular surface reconstruction applications [174]. Crosslinked AM showed higher interconnectivity among collagen fibres and promoted in vitro cell growth and angiogenesis, without eliciting an immune response [174].

TGs crosslinking has also been used in bone grafting, for example, to enhance interfacial adhesion of collagen/osteopontin on mineral substrates, increase the fracture toughness of bone [175,176], and produce collagen/nano-hydroxyapatite/chondroitin sulfate scaffolds, with possible clinical applications for spinal fusion surgery [177]. Collagen-based biomaterials crosslinked by TG2 have shown to increase cellular response in bone healing, by promoting the expression of integrins in human osteoblasts [178].

**Figure 4:**
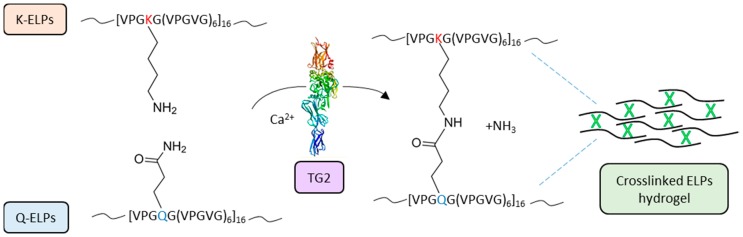
Schematic representation of TG2-crosslinked ELPs hydrogels. The repeat sequence [VPGXG(VPGVG)_6_]_16_ of ELPs was genetically designed to produce two libraries by substituting the X residue with Lys (K) and Gln (Q), thus generating K-ELPs and Q-ELPs, respectively. TG2 mediates the formation of a crosslink between the Gln and Lys residues of ELPs [191]. The Q and K side chains are superimposed to visualize the TG reaction. TG2 three-dimensional structure (PDB: 2Q3Z) was produced with the molecule modelling software, “EzMol” [34].

Similarly, other natural biopolymers, such as cellulose, fibrin, alginate, and hyaluronic acid (HA), have been used for the production of both tissue engineering scaffolds and drug delivery matrices [179,180,181,182,183,184] and, in the last 20 years, also synthetic peptide-based biopolymers have been exploited. In particular, elastin-like polypeptides (ELPs) hydrogels have been applied in multiple medical procedures, like cartilage and intervertebral disc tissue repair, vascular grafts, stem cell matrices, and post-surgical wound treatment to mention a few [185,186,187,188,189,190]. McHale and colleagues designed Lys- and Gln-containing ELPs by substituting the residue in position X of the ELP repeat sequence, VPGXG(VPGVG)_6_, with Lys and Gln, respectively (Figure 4). These ELPs can be crosslinked by TG2 in a biocompatible process and form hydrogels able to encapsulate chondrocytes, leading to an increased ECM deposition and mechanical integrity [191].

Interestingly, TG2 had already been tested as a biocompatible glue used to join two articular cartilage pieces in 1997 by Jürgensen, who demonstrated that TG2 treatment increased the adhesive strength by 40%, displaying a better performance compared to a commercial tissue sealant [192].

Recently, TG2 has been used to substitute the chemical agent, tris(hydroxylmethyl)phosphine (THP), for the production of resilin-based (RZ) protein gels, with applications in tissue engineering. TG-produced RZ matrices were more suitable for long-term cell attachment compared to those formed with THP. Moreover, as TG-derived matrices mimic the subendothelial environment more successfully compared to hard glass surfaces, they provide a more suitable environment for endothelial differentiation in vitro [193].

TGs have also shown promise for the fabrication of biopolymer microgels, small particles composed by crosslinked polymers that form three-dimensional (3D) structures filled with water, and are thus especially suitable for the delivery of nutrients and bioactive molecules [194,195,196,197]. One application of TGs-produced 3D hydrogels is in neuro tissue-engineering. In this context, the most commonly used hydrogels are made by chemical crosslinking of high molecular weight HA, which is an essential component of the central nervous system’s ECM, with anti-inflammatory and anti-fibrotic properties, using agents, such as 1,4-butanediol diglycidyl ether (BDDE), adipic dihydrazide (ADH), or ethyl N, N-dimethylaminopropyl carbodiimide (EDC) [198,199]. The use of FXIIIa, able to crosslink HA modified by the addition of TG substrate peptides providing a reactive Gln or Lys residue (HA-TG), has been shown to allow the formation of better HA-based hydrogels compared to the chemically produced ones [200]. In fact, these hydrogels present higher chemical stability, more specific crosslinking, and features, such as tuneable gelation speed and stiffness, cytocompatibility, and injectability. Furthermore, HA-TG hydrogels can create covalent crosslinks with fibrin and other proteins, and can be a target of enzymatic degradation, which facilitates bioresorption [200].

Poly-ethyl-glycol (PEG) polymers are among the most commonly used molecules for the development of drug-delivery systems, as they are biocompatible and easily modified to form hydrogels. Indeed, these hydrogels can be produced by TGs crosslinking when functionalised with peptides, then mixed with therapeutic agents and potentially injected in the body. For example, TG2 was able to form highly elastic hydrogels in less than two minutes by creating crosslinks between the Gln residue of a PEG containing Ac-GQQQLG-NH_2_ and the Lys residue of a PEG containing DOPA-FKG-NH_2_ [201,202]. These hydrogels were tested as tissue glues on both guinea pig skin and collagen membranes, showing similar and higher adhesive strength, respectively, compared to fibrin tissue sealants [202]. Besides this application, PEGs have also been largely used in bio-conjugation processes.

#### 4.2.2. Bio-Conjugation

PEGs conjugation of therapeutic proteins, defined as PEGylation, has been used to decrease proteins’ immunogenicity, which is relevant for the improvement of the pharmacological proprieties of drugs [203,204]. By exploiting TGs’ requirements for sequence and structure specificity for the targeting of Gln residues (amine acceptor site) [205], different groups have shown that classical random conjugation of PEG on proteins, which produces heterogeneous results, can be replaced by the more efficient TGs-dependent PEGylation (by mTG and FXIIIa), which allows the production of single site-specific conjugate isomers and, at the same time, the preservation of the proteins’ bioactivities [206,207,208,209,210]. Interestingly, Sato et al. showed that the mTG-mediated incorporation of site-specific alkylamine-PEG conjugates into recombinant human interleukin-2 (rhIL-2), acting as the Gln donor substrate, did not affect rhIL-2 bioactivity, as opposed to random derivatisation. Moreover, pharmacokinetics studies in rodents revealed that the conjugates presented an increased half-life (up to 6-fold) compared to unmodified rhIL-2 [206]. mTG has also been used in vitro for protein lipidation, in order to increase the protein-lipid conjugate amphiphilicity and thus control its localisation at natural or artificial membranes’ interfaces [211].

**Figure 5:**
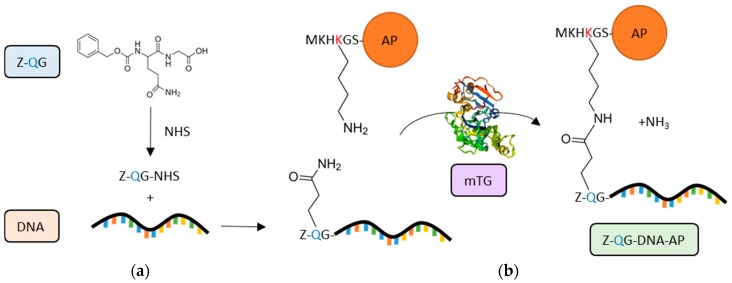
Protein-DNA conjugation. By exploiting benzyloxycarbonyl-l-glutaminylglycine (Z-QG) as a TG substrate, mTG induces site-specific and covalent conjugation of DNA to proteins, such as alkaline phosphatase (AP). (**a**) Chemical activation of Z-QG carboxylate with N-hydroxysuccinimide (NHS) and formation of the activated Z-QG-NHS, which is then modified by addition of an aminated oligodeoxynucleotide (DNA). (**b**) mTG mediates the formation of a crosslink between the Gln residue of Z-QG-DNA and the Lys residue of the MKHKGS peptide tag fused to AP. The Q and K side chains are superimposed to visualize the TG reaction [213]. The modelling software, “EzMol”, was used to generate mTG structure (PDB: 1IU4) [34].

An alternative TGs substrate used for bio-conjugation is benzyloxycarbonyl-l-glutaminylglycine (Z-QG), already well-known in TG activity assays [212]. A specific application of Z-QG tags is the creation of protein–oligonucleotide (DNA) conjugates, which are useful tools in molecular biology, in particular for the production of protein microarrays. In order to overcome some issues related to the chemical manipulation commonly used for DNA-directed immobilisation, mTG has been successfully used to induce site-specific and covalent conjugation of DNA to peptide tags [213]. Specifically, mTG was able to mediate the labelling of K6-tagged recombinant proteins (K6 = MKHKGS), i.e., alkaline phosphatase (AP) and enhanced green fluorescent protein (EGFP), to an aminated DNA coupled with Z-QG, forming a protein-DNA conjugate (Z-QG-DNA tagged proteins) (Figure 5) [213]. 

An improvement of the same approach was explored for the sensitive and cost-effective preparation of Z-QG-DNA–AP conjugates for filter and in situ hybridisation assays [214]. Moreover, this procedure was used to functionalise RNA (Z-QG-RNA-AP conjugates) and tested in tissue sections by in situ hybridisation [215]. The Z-QG conjugation approach has been broadened to the production of DNA aptamer-(protein)_n_ conjugates for cell imaging through a two-step reaction mediated by terminal deoxynucleotidyl transferase (TdT) and mTG [216,217]. These biocompatible mTG-derived constructs offer novel opportunities for the development of non-invasive in vivo imaging [217].

The successful use of TGs for the creation of bio-conjugates has led to the application of this procedure for protein fluorescent labelling and immobilisation. Keillor and colleagues demonstrated that mTG can be used for the site-specific labelling of proteins genetically modified with encodable high-affinity Gln-substrates (‘Q-tags’) through incorporation of propargylamine into the Gln residues (propargylation) [218,219]. This strategy showed high potential for the conjugation of a wide range of azide derivatives for fluorescence labelling, with possible applications in living cells [219,220,221].

**Figure 6:**
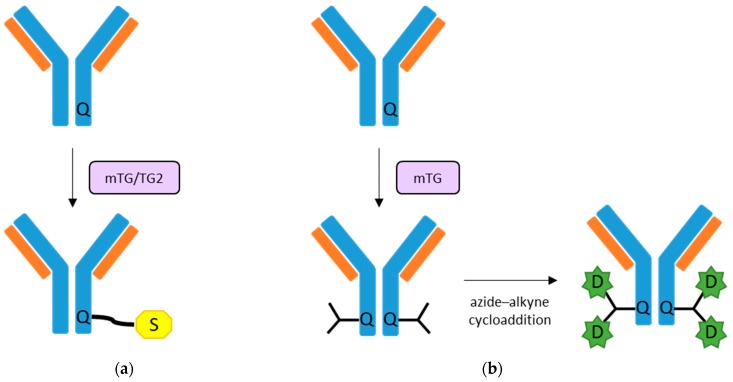
Schematic representation of TG-mediated antibody conjugation. (**a**) TGs can transamidate modified substrates (e.g., aminated or Lys carrying derivatives), such as biotin, fluorophores, or radioisotopes (schematically shown with the yellow “S”), to a carboxamide group of a Q residue on the antibody heavy chain peptide sequence [222,223,225]. (**b**) Branched linkers, conjugated by mTG to the heavy chain of an antibody (e.g., anti-HER2 monoclonal antibody), can be coupled by azide-alkyne cycloaddition to an antimitotic drug (shown as a green “D”) [224].

Notably, antibodies are among the proteins that can be functionalised by TGs for diagnostic and therapeutic purposes (Figure 6). Josten and colleagues proposed an original enzymatic biotinylation method useful for the production of low-biotinylated proteins, based on mTG-mediated incorporation of amino-modified derivatives on IgG Gln residues [222]. More recently, antibody functionalisation by TGs has been applied in radio immunodiagnosis and therapy antibody [223,224,225]. Both mTG and, to a lesser extent, human TG2 have shown the ability to perform the selective modification of antibodies heavy chains (IgGs), without interfering with their biological activities, such as antigen affinity and cell internalisation, as tested both in vitro and in vivo [223,225]. Interestingly, a new multi-loading approach that improves the drug-to-antibody ratio has been tested, with promising applications in targeted therapy [224]. Specifically, mTG conjugation of branched linkers on the heavy chain of an anti-HER2 monoclonal antibody have shown to increase the drug cytotoxicity against a HER2-expressing breast cancer cell line in vitro, compared to conjugates carrying the classic linear linkers [224].

## 5. Conclusions

There is no question that TG-biocatalysis is instrumental in determining protein multimerisation, and that several are the natural substrates of TG, which can be permanently modified by transamidation. Although research has documented numerous physio-pathological conditions in which TG family members are involved, there is still much to be learnt about the way the catalytic activity of these enzymes is controlled in vivo. In parallel, there is a great interest in the application of transglutaminases, especially mTG and TG2, as a tool to catalyse the formation of amide bonds between peptide or protein bound glutamines and lysines, or to change protein proprieties via incorporation of polyamines in a variety of uses of particular relevance to the biomedical and food industry. Even though substrate specificity remains a challenge, the versatility and biocompatibility of transglutaminases continue to make them attractive for a wide range of biotechnological applications. 

## Figures and Tables

**Table 1:** List of applications involving TGs activity.

Applications	References
**Food Industry**	
Dairy products (caseins and whey proteins)	[118,120,135,136,137]
Soybean proteins	[121]
Bakery products (gluten)	[122,123,138,139,140,141,142]
Meat (myosins and myofibrillar protein)	[124,125,129,130,131,132,133,134]
Eggs (ovalbumin)	[126]
Seafood and edible films	[127,128,147,148,149,150,151,152,153]
**Biomedicine**	
Collagen-based scaffolds and hydrogels	[70,73,74,161,162,163,166,167,169,170,171,172,173,174,175,176,177,178]
Other natural biopolymers-based hydrogels and microgels (Fibrin, gelatin, alginate, hyaluronic acid, casein)	[179,180,181,182,183,184,194,195,197,198,199,200]
Synthetic biopolymers-based scaffolds and 3D microgels (ELPs, RZ, PEG)	[189,190,191,193,196,201,202]
PEGylation/Lipidation	[206,207,208,209,210,211]
Protein-DNA conjugation	[213,214,216,217]
Protein fluorescent labelling	[218,219,220,221]
Antibodies functionalization	[223,224,225]
